# Quality evaluation of health science popularization short videos related to cerebrovascular diseases on popular short video platforms in China: cross-sectional study

**DOI:** 10.3389/fpubh.2026.1764220

**Published:** 2026-02-19

**Authors:** Xingyu Liu, Xueping Jiao, Mengting Liu, Shuhan Yang, Yueting Wang, Xueqin Yang, Yuhuan Xie, Yufang Guo, Fanghong Yan, Yuxia Ma, Junxia Wang, Yanan Zhang

**Affiliations:** 1 School of Nursing, Lanzhou University, Lanzhou, Gansu Province, China; 2 Department of Neurosurgery, Gansu Provincial People's Hospital, Lanzhou, Gansu Province, China; 3 Department of Geriatrics, Gansu Third Provincial People's Hospital, Lanzhou, Gansu Province, China

**Keywords:** health science popularization, short videos, cerebrovascular diseases, TikTok, Kuaishou

## Abstract

**Background:**

Health science popularization short videos have become one of the main sources of acquiring disease-related information. However, the quality of such videos on popular short video platforms varies considerably. This study aims to evaluate the quality of the health science popularization short videos about cerebrovascular diseases on two popular short video platforms (TikTok and Kuaishou) in China.

**Methods:**

Using Python web crawler, short videos related to cerebrovascular diseases were collected from TikTok and Kuaishou in China, posted from December 10th, 2023, to December 10th, 2024. Ultimately, 915 valid videos were included. Two clinical experts evaluated the quality of the included videos using GQS, mDISCERN, and PEMAT-A/V independently. The median (IQR) was used to describe the features of the short videos, and the *Kruskal-Wallis* test was used to evaluate the differences between groups. Correlation analysis and the Random Forest regression model were applied to investigate the correlation between the features and the quality score of short videos.

**Results:**

Health science popularization short videos related to cerebrovascular diseases on the TikTok platform showed significantly more likes, favorites, comments, and shares (*p* < 0.001). The videos on TikTok had a median score of 3 on mDISCERN, a median score of 3 on GQS, a median Understandability score of 65.38%, and a median Actionability score of 50%, all of which were significantly higher than those on Kuaishou. There were strong correlations between video duration and mDISCERN score (*r* = 0.219, *p* < 0.001), GQS (*r* = 0.495, *p* < 0.001), Understandability score (*r* = 0.282, *p* < 0.001), and Actionability score (*r* = 0.361, *p* < 0.001). The four Random Forest regression models for video quality scores demonstrated favorable fitting performance, with *R*^2^ values ranging from 0.862 to 0.903.

**Conclusion:**

Health science popularization short videos related to cerebrovascular diseases on TikTok and Kuaishou showed a moderate quality, and the quality of the health science popularization short videos on TikTok was better than those on Kuaishou. Video duration was a key determinant of video quality.

## 1 Introduction

Cerebrovascular diseases, also known as cerebrovascular accidents (CVA), pose a significant threat to public health with the rising prevalence (1, 2). The WSO-*Lancet Neurology* Commission on Stroke predicted that from 2020 to 2050, the global stroke mortality rate would increase by 50%, and the DALYs would increase from 144.8 million in 2020 to 189.3 million in 2050 (3). In China, approximately 3.94 million new stroke cases occur annually, accounting for one-third of the total number of global (4). Sufficient health literacy is critical for the prevention, treatment, and post-rehabilitation management of cerebrovascular diseases (5). However, the public’s literacy on cerebrovascular diseases remains insufficient, particularly in rural areas, where insufficient disease related literacy leads unhealthy health behaviors, which in turn causes poor prognosis (6). The WSO-*Lancet Neurology* Commission on Stroke recommended that improving the literacy of the public through mobile and digital technologies (7). The “*Healthy China 2030*” *Planning Outline* pointed out that health science popularization, as an important means to enhance national health literacy and achieve Healthy China goals, required the media to strengthen health science dissemination and promotion to comprehensively improve national health (8). Therefore, it is necessary to popularize health knowledge to the public through internet, thereby improving their knowledge reserves about cerebrovascular diseases and supporting informed health behaviors.

Short video platforms have become the dominant channels for health knowledge dissemination and 80% of Internet users search medical information through short video platforms (9). By December 2024, the number of short video users in China had reached 1.04 billion, accounting for 97.6% of the total netizen population (10). Compared with traditional medical books and newspapers, health science popularization short videos could disseminate health information with a more understandable way about healthy diet and lifestyle, vaccination, rational drug use and disease prevention (11). Moreover, short videos could spread disease-related knowledge more effectively with wide dissemination, easy accessibility, easily understandable content, and the ability to interact with the audience at any time (12). However, the quality of health science popularization videos on short video platforms varies and need to be evaluated comprehensively.

Studies reported that health science popularization videos with low quality usually contained disinformation and misinformation (9). During the COVID-19 pandemic, it was precisely low-quality health content—such as videos containing misinformation and inaccurate details about vaccines—that spread widely on social media, thereby fostering public distrust in public health measures (13). Li (14) revealed that only 11% of COVID-19 videos on YouTube were high-quality with authentic content posted by governments and professionals, while 28% of them contained non-factual and misleading information. Shockingly, these inaccurate videos amassed a staggering 62 million views. Similar issues persist in low-quality health science popularization short videos covering topics like smoking and bladder cancer, with millions of views (15, 16). Such low-quality health information not only impairs the public’s ability to make informed health decisions but also hinders the effective implementation of public health policies (17, 18). For example, Lia (19) reported that low-quality health science popularization videos about *Helicobacter pylori* may lead patients to make incorrect judgments regarding disease management. Therefore, evaluating the quality of health science popularization videos on short video platforms could provide a basis for the short video platform and government to manage and restrict the dissemination of low-quality health science popularization short videos.

TikTok and Kuaishou are the two leading short video platforms with the highest market share in China, boasting a massive online user base and facilitating public access to health information. Quest Mobile data showed that as of March 2025 (20), the monthly active user scale of China’s mobile Internet has reached 1.259 billion, with the Chinese versions of TikTok and Kuaishou platforms accounts for nearly 70% of the market. Although health-related content on foreign platforms such as TikTok and YouTube has been evaluated in several studies (21–23), few studies focused on China’s TikTok and Kuaishou. Moreover, certain studies have employed only a single evaluation tool, failing to comprehensively assess the quality of short videos.

Therefore, the primary objective of this study was to systematically evaluate the quality of health science popularization short videos on TikTok and Kuaishou platforms using multiple assessment tools, as well as to identify the key factors influencing the video quality. Our research will help general public distinguish health science popularization videos of different qualities on short video platforms, enabling them to access accurate and comprehensive disease-related health knowledge. Additionally, it will provide new insights for governing low-quality science popularization videos.

## 2 Methods

### 2.1 Search strategy

**Figure 1:**
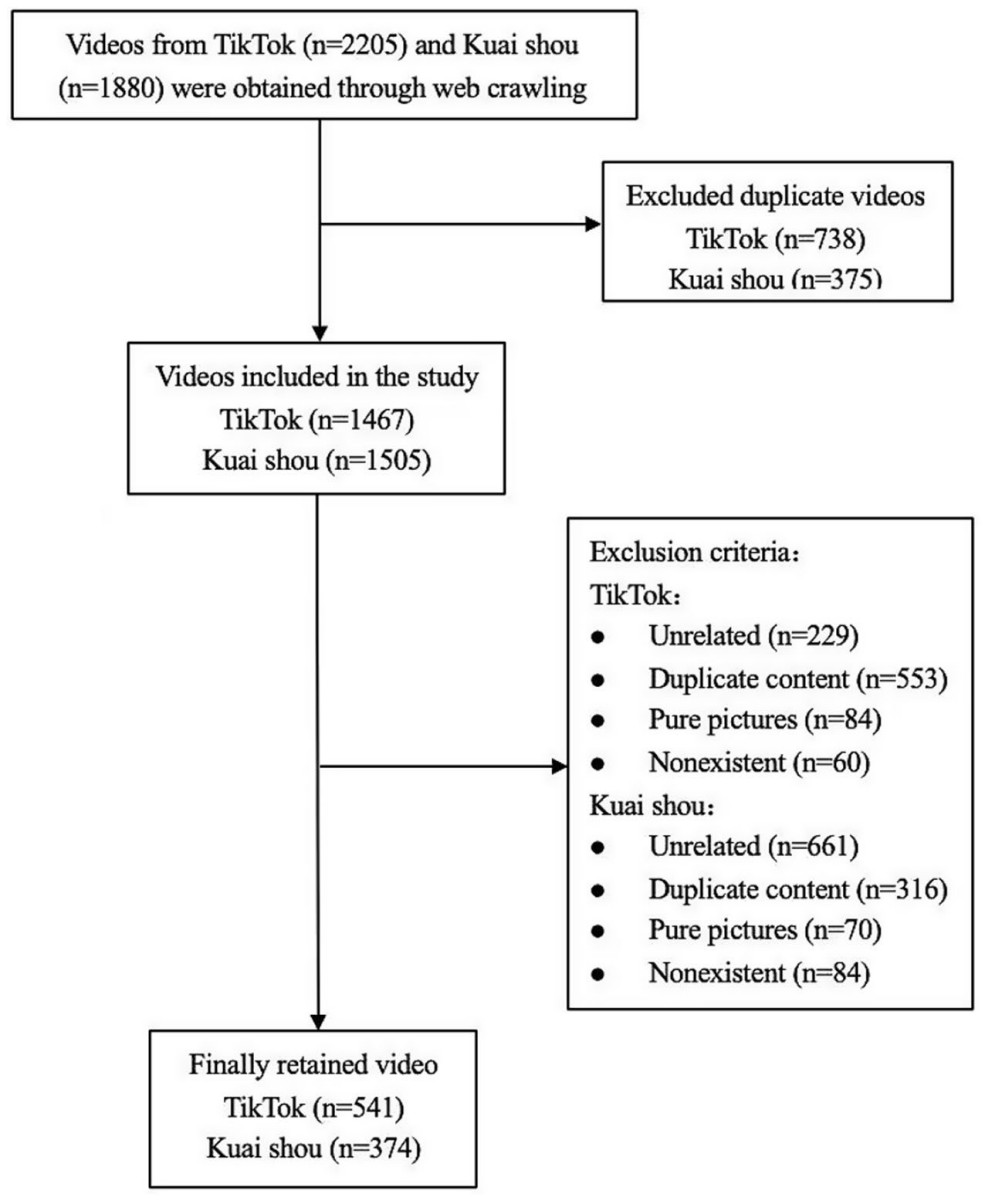
Search strategy of health science popularization short videos related to cerebrovascular diseases on TikTok and Kuaishou.

Using Python web crawling technology, we scraped short videos related to seven Chinese keywords on the Chinese versions of TikTok and Kuaishou: “cerebrovascular disease,” “cerebral apoplexy,” “stroke,” “cerebral infarction,” “transient ischemic attack”, “intracerebral hemorrhage”, and “subarachnoid hemorrhage.” The crawler employed the DrissionPage library for browser automation and network request monitoring, the time library to set random time for simulating human browsing behavior, and a custom DataRecorder module for data logging. The crawling frequency was configured such that a random wait time of 2–4 s was imposed after each page scroll, with 3–5 random scrolls performed per crawling cycle. The formulation of keywords was based on the standard terminology specified in the *Guidelines for the Prevention and Treatment of Cerebrovascular Diseases (2024 Edition)* issued by the *National Health Commission of the People’s Republic of China* (4), as well as commonly-used public search terms, covering the main subtypes of cerebrovascular diseases. We included all videos related to the keywords published between December 10th, 2023, and December 10th, 2024. A total of 2,205 videos from TikTok and 1880 videos from Kuaishou were retrieved using 7 keywords. After merging and removing duplicates of videos related to different diseases, 1,467 TikTok videos and 1,505 Kuaishou videos remained. Following manual screening to exclude videos unrelated to health science popularization, duplicate content, pure picture videos, and nonexistent videos, a final total of 541 TikTok videos and 374 Kuaishou videos were included in this study (Figure 1).

### 2.2 Data collection

Basic information of the videos, including video title, type of account, video duration, number of likes, number of favorites, number of comments, and number of shares, was extracted. For data missing from the web crawling process, manual video retrieval was performed to supplement the dataset. In this study, the quality of the video content was evaluated, while the quality of the video images and music was not assessed in the study. According to the authentication information displayed on the platforms (such as official certification badges, profile descriptions, and verified qualifications), account types were categorized into the following categories with specific definitions to facilitate subsequent processing: (1) Medical professional accounts: Accounts authenticated with credentials of medical practitioners, with profile information clearly indicating their professional identity (for example, “neurologist at XX Hospital”); (2) Hospital accounts: Official accounts registered by medical institutions with the full name and affiliation of the hospital in their profiles, such as hospitals, clinics, or medical centers; (3) Government accounts: Accounts operated by government agencies related to public health, such as health commissions and centers for disease control and prevention; (4) Media accounts: News entities with legal news qualifications, such as Yangshipin (China Media Group’s online platform) and XX City Radio and Television Station; (5) Company accounts: Accounts registered by enterprises or commercial entities, such as pharmaceutical companies and health product brands; (6) Non-professional individual accounts: Personal self-media accounts without official certification of medical, institutional, or media qualifications.

### 2.3 Quality assessment

The quality of the included short videos was scored using the Global Quality Score (GQS), modified DISCERN (mDISCERN) and the Patient Education Materials Assessment Tool (PEMAT) to perform a comprehensive assessment of the included videos. The GQS was employed to assess the overall quality of included short videos. The 5-point GQS was developed by Bernard et al. in 2007 (24). Singh et al. first applied it to video assessment in 2012 (25), and it has now been widely adopted for this purpose (26–28). The total score ranges from 1 to 5, with lower scores indicating poorer video quality and higher scores representing superior quality. The specific scoring details for GQS are shown in Supplementary Table 1. The mDISCERN was used for reliability evaluation. The DISCERN criteria are a validated scoring system developed by a research team at the University of Oxford to assess the information quality and reliability of health content related to consumer treatment options. The Cronbach’s *α* coefficient was 0.78 (29). The mDISCERN tool, modified by Singh et al. (25), is more suitable for evaluating videos (30). It consists of five questions, each question was scored 1 for “yes” and 0 for “no,” and high scores indicated that the video was reliable. The specific scoring details for mDISCERN are shown in Supplementary Table 2. The PEMAT was adopted for assessing the comprehensibility and operability of the included short videos. The PEMAT-A/V (31), designed specifically for audiovisual materials, consists of 17 questions, with 13 questions that evaluate the understandability of health information received by patients and 4 questions evaluating the actionability of recommendations by videos. The Cronbach’s *α* coefficient was 0.71 (31). Each question is scored as “agree = 1, disagree = 0, not applicable = N/A”, and the score of the understandability or actionability section is calculated as “Total Points/Total Possible Points×100%”, with higher scores indicating better performance in terms of understandability and/or actionability of the video. The specific scoring details for PEMAT-A/V are shown in Supplementary Table 3. Two clinical experts independently used GQS, mDISCERN and PEMAT-A/V to evaluate the quality of health science popularization short videos about cerebrovascular diseases on short video platforms. Before accessing the short videos, the assessment criteria of each scoring tool were reviewed by the two experts, and a detailed introduction about the three evaluation tools was given to the two experts to reduce the errors caused by cognitive biases. The scoring process was independently conducted by two clinical experts, and the final quality score of each video was determined by taking the average of their respective ratings.

### 2.4 Statistical analyses

**Figure 2:**
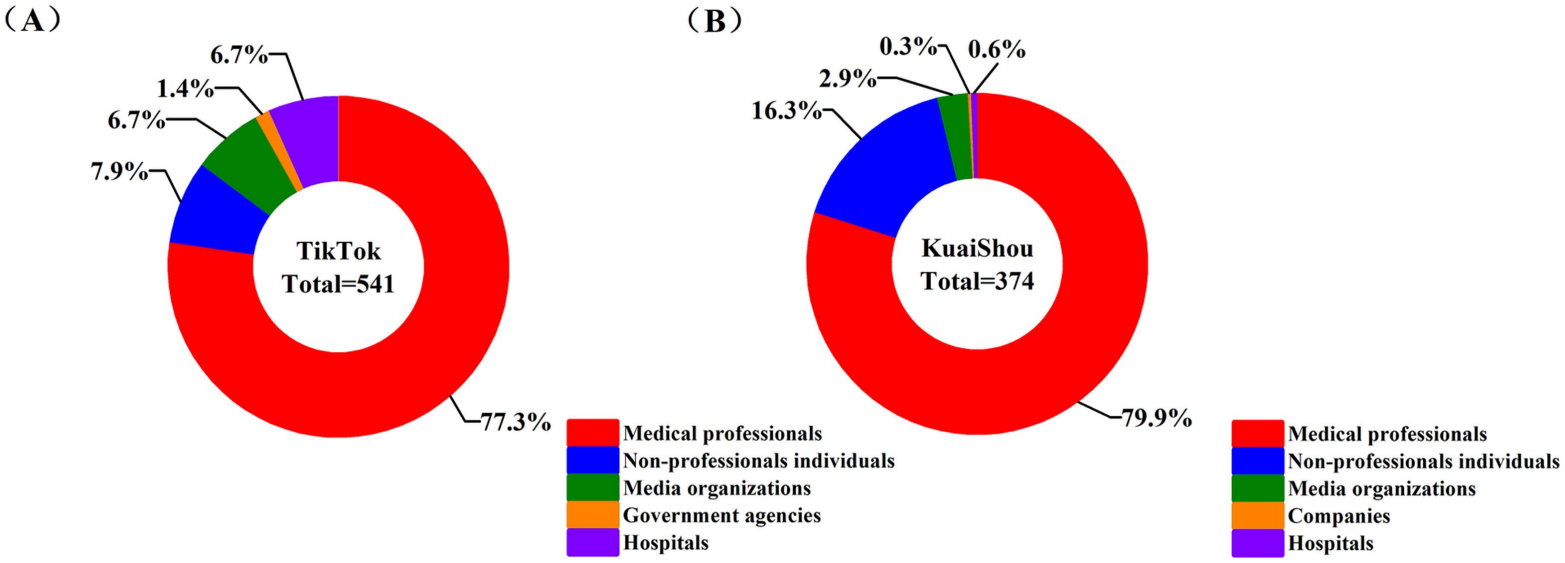
Distribution of different account types. **(A)** Distribution of different account types on TikTok; **(B)** Distribution of different account types on Kuaishou.

All data were double-checked. SPSS 27.0 was used for statistical analysis, and Origin 2024 was used for graphing. The consistency evaluation of the scoring results of the two raters was carried out by using the Intraclass Correlation Coefficient (ICC). Since the data were nonparametrically distributed, the median (IQR) was used for the descriptive statistics. The *Kruskal-Wallis* test was used to assess the differences between groups. And Spearman correlation analysis was used to evaluate the relationship between the video features and quality score of videos. The significance of the statistical analysis was set as 0.05. The Random Forest regression model was used to examine the degree of importance of video features on video quality. The initial parameters were set with the number of decision trees at 100, all other parameters were kept at their default values, and 5-fold cross-validation was adopted to optimize the model parameters (Figure 2A).

## 3 Results

### 3.1 Basic features of health science popularization short videos related to cerebrovascular diseases on TikTok and Kuaishou

We retained 915 health science popularization short videos related to cerebrovascular diseases in this study, including 541 from TikTok and 374 from Kuaishou. The videos were published by accounts with different entities. On TikTok, medical professionals released the highest number of videos, accounting for 77.3% (418/541), followed by non-professional individuals (43/541, 7.9%), media organizations (36/541, 6.7%), hospitals (36/541, 6.7%) and government agencies (8/541, 1.4%) (Figure 2A). Similarly, on Kuaishou, medical professionals released the highest number of videos, accounting for 79.9% (299/374), followed by non-professional individuals (61/374, 16.3%), media organizations (11/374, 2.9%), hospitals (2/374, 0.6%) and companies (1/374, 0.3%) (Figure 2B). The number and proportion of different keywords retrieved from the two platforms were detailed in Supplementary Table 4. The results showed that videos related to “cerebrovascular disease” accounted for the largest proportion (241/915, 26.3%), followed by those related to “cerebral apoplexy” (201/915, 22.0%), and videos related to “subarachnoid hemorrhage” constituted the smallest proportion (76/915, 8.3%).

**Figure 3:**
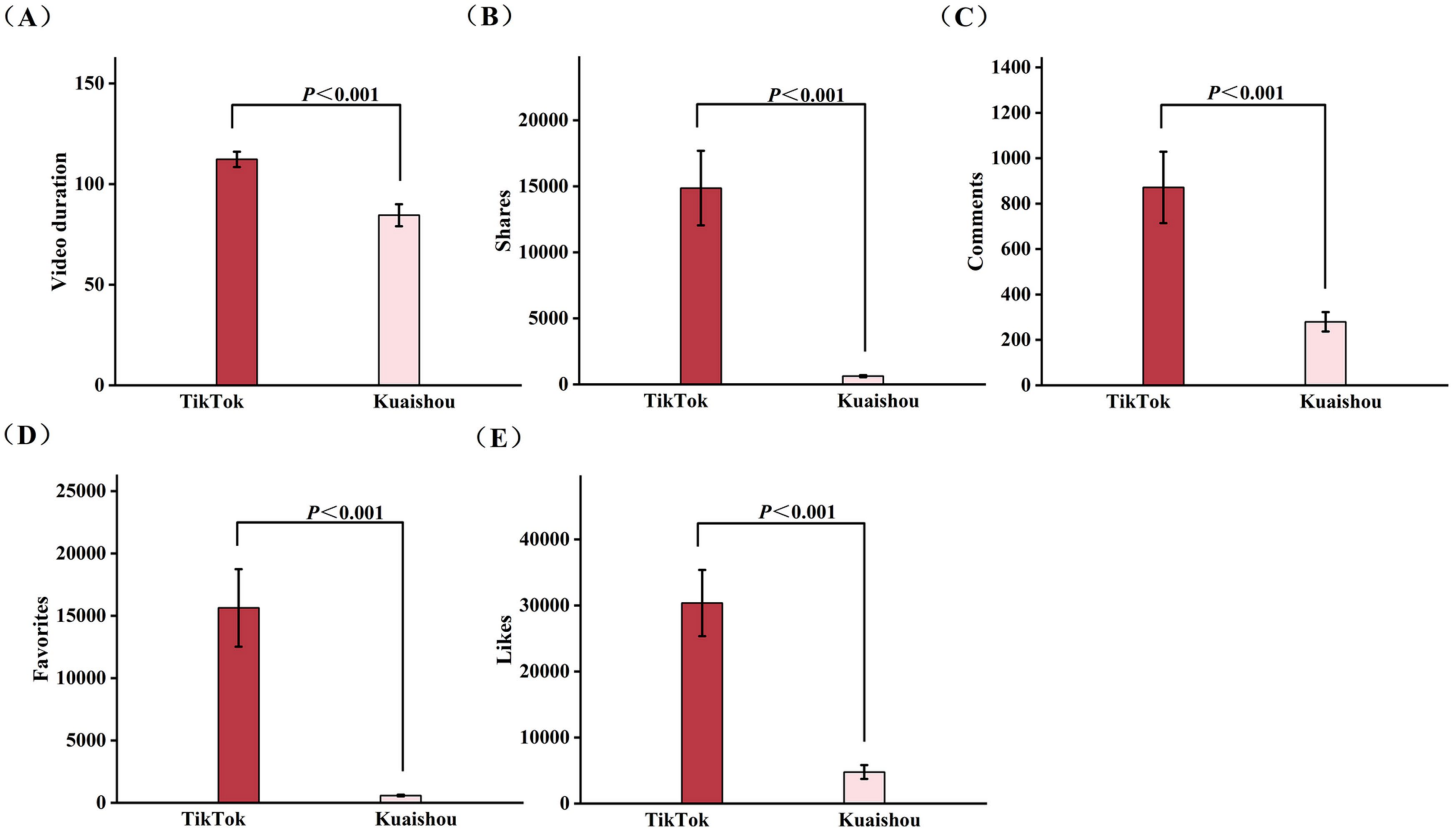
Comparison of the video features of cerebrovascular diseases health science popularization short videos on TikTok and Kuaishou. **(A)** Comparison of likes of cerebrovascular diseases health science popularization short videos on TikTok and Kuaishou; **(B)** Comparison of favorites of cerebrovascular diseases health science popularization short videos on TikTok and Kuaishou; **(C)** Comparison of shares of cerebrovascular diseases health science popularization short videos on TikTok and Kuaishou; **(D)** Comparison of comments of cerebrovascular diseases health science popularization short videos on TikTok and Kuaishou; **(E)** Comparison of video duration of cerebrovascular diseases health science popularization short videos on TikTok and Kuaishou.

Furthermore, we conducted a comparative analysis of TikTok and Kuaishou short videos across five key features: video duration, likes, favorites, comments, and shares (detailed in Table 1). The results showed that the TikTok videos got more likes, favorites, comments, shares than those on Kuaishou (*p* < 0.001). The median video duration on Tiktok was 89 (58–140) seconds, was significantly longer than those on Kuaishou, which was 68 (42–101) seconds (*p* < 0.001). A detailed comparative analysis of these video features was presented in Figure 3.

**Table 1:** Comparison of video features of cerebrovascular diseases health science popularization short videos on TikTok and Kuaishou.

Features	TikTok(*n* = 541)	Kuaishou(*n* = 374)	*Z*	*p*
	Median (IQR)	Median (IQR)		
Duration	89(4–742)	68(4–1,495)	6.519	<0.001***
Likes	520(118–10,653)	190(28–896)	6.976	<0.001***
Comments	34(8–344)	18(2–93)	4.251	<0.001***
Shares	127(16–3,537)	70(10–424)	3.815	<0.001***
Favorites	131(22–3,743)	63(7–353)	5.388	<0.001***

***p < 0.001.

### 3.2 Quality of health science popularization short videos related to cerebrovascular diseases on TikTok and Kuaishou

**Figure 4:**
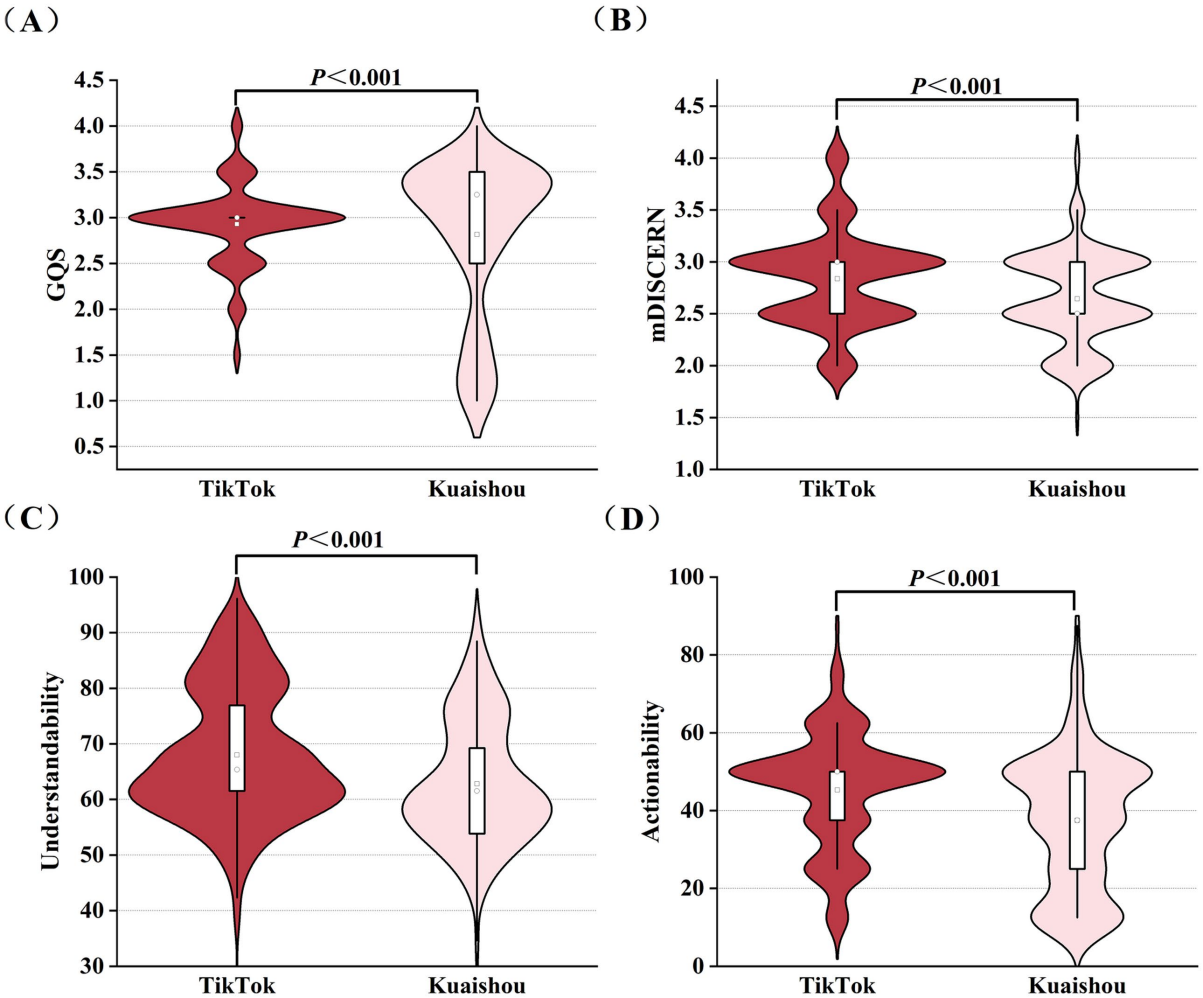
Comparison of the cerebrovascular diseases health science popularization short videos on TikTok and Kuaishou in different score ranges across different tools. **(A)** Distribution of the mDISCERN scores of cerebrovascular diseases health science popularization short videos on TikTok and Kuaishou; **(B)** Distribution of the GQS of cerebrovascular diseases health science popularization short videos on TikTok and Kuaishou; **(C)** Distribution of the Understandability scores of cerebrovascular diseases health science popularization short videos on TikTok and Kuaishou; **(D)** Distribution of the Actionability scores of cerebrovascular diseases health science popularization short videos on TikTok and Kuaishou.

**Figure 5:**
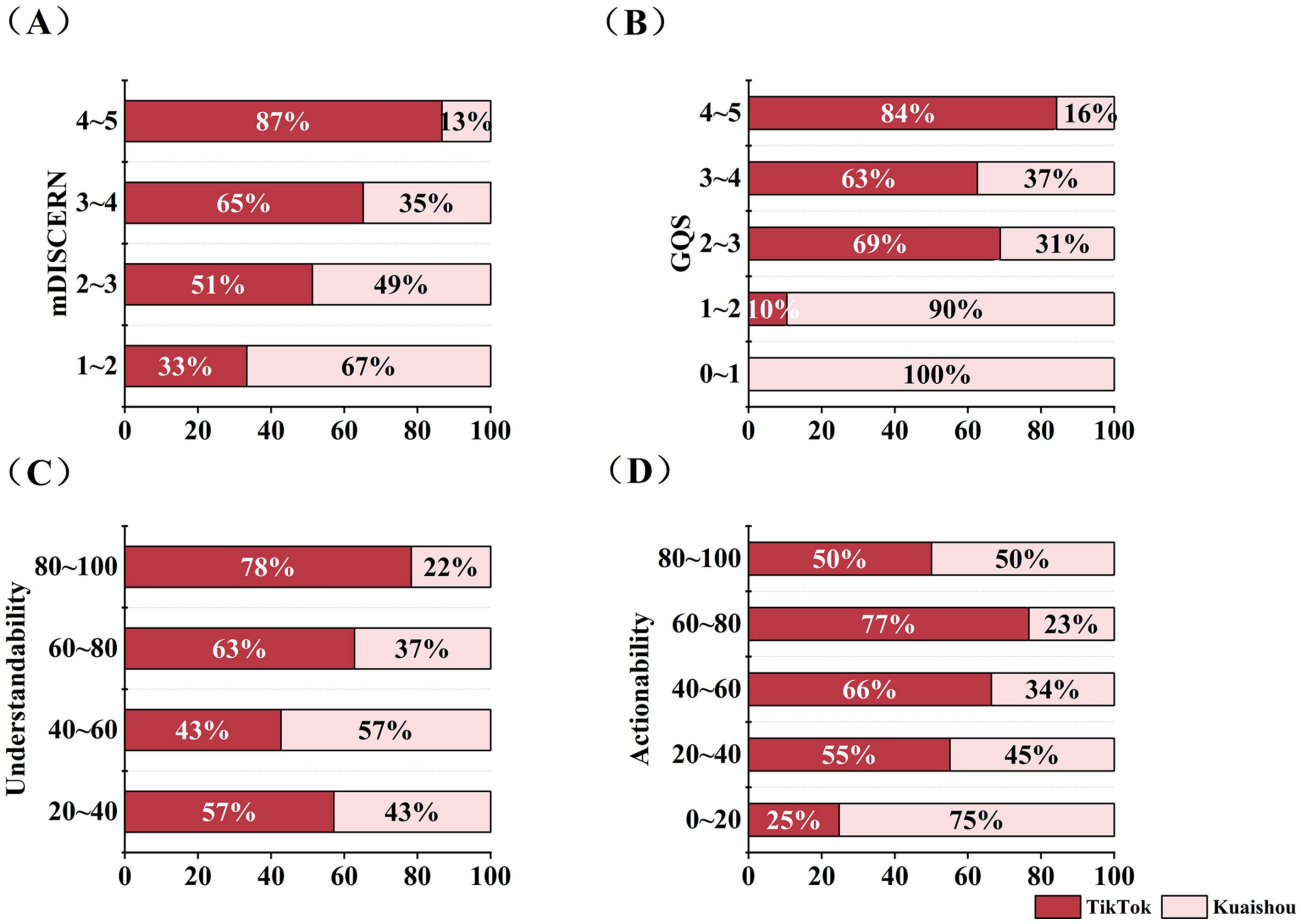
Quality scores of cerebrovascular diseases health science popularization short videos on TikTok and Kuaishou. **(A)** GQS of cerebrovascular diseases health science popularization short videos on TikTok and Kuaishou; **(B)** mDISCERN scores of cerebrovascular diseases health science popularization short videos on TikTok and Kuaishou; **(C)** Understandability scores of cerebrovascular diseases health science popularization short videos on TikTok and Kuaishou; **(D)** Actionability scores of cerebrovascular diseases health science popularization short videos on TikTok and Kuaishou.

The consistency between the two raters was satisfactory, with ICC ≥ 0.7, details were shown in Table 2. The certain differences in the video quality scores across different keywords were detailed in Supplementary Table 4. The median Understandability score for “cerebrovascular disease videos” was the highest, at 65.38% (IQR: 57.69–80.77%), and the median Actionability score for “transient ischemic attack” and “subarachnoid hemorrhage” videos was the lowest, both at 25% (IQR: 25–50%). The mDISCERN and GQS scores of videos associated with different keywords were essentially consistent. Regarding videos on TikTok, the median mDISCERN score was 3 (IQR 2.5–3), the GQS median score was 3 (IQR 3–3), the median Understandability score was 65.38% (IQR 61.54–76.92%) and the median Actionability score was 50% (IQR 37.5–50%), indicating that the videos on TikTok were of fair quality. Regarding Kuaishou videos, the median mDISCERN score was 2.5 (IQR 2.5–3), the GQS median score was 3.25 (IQR 2.5–3.5), the median Understandability score was 61.54% (IQR 53.85–70.19%) and the median Actionability score was 37.5% (IQR 25–50%). The comparison of scores between TikTok and Kuaishou videos across different score ranges evaluated by various tools was shown in Figure 4. The results demonstrated that TikTok videos achieved significantly higher scores than Kuaishou across all video quality metrics, including the mDISCERN scores, 5-level GQS, Understandability, and Actionability (Figure 5).

**Table 2:** Internal consistency between the two raters.

Platform	Scale	ICC	95%CI
TikTok	mDISCERN	0.740	0.693–0.781
	GQS	0.831	0.800–0.858
	Understandability	0.877	0.855–0.896
	Actionability	0.896	0.877–0.912
Kuaishou	mDISCERN	0.798	0.753–0.835
	GQS	0.858	0.827–0.884
	Understandability	0.850	0.816–0.878
	Actionability	0.852	0.819–0.880

ICC, Intraclass Correlation Coefficient; mDISCERN, modified DISCERN; GQS, Global Quality Score.

**Figure 6:**
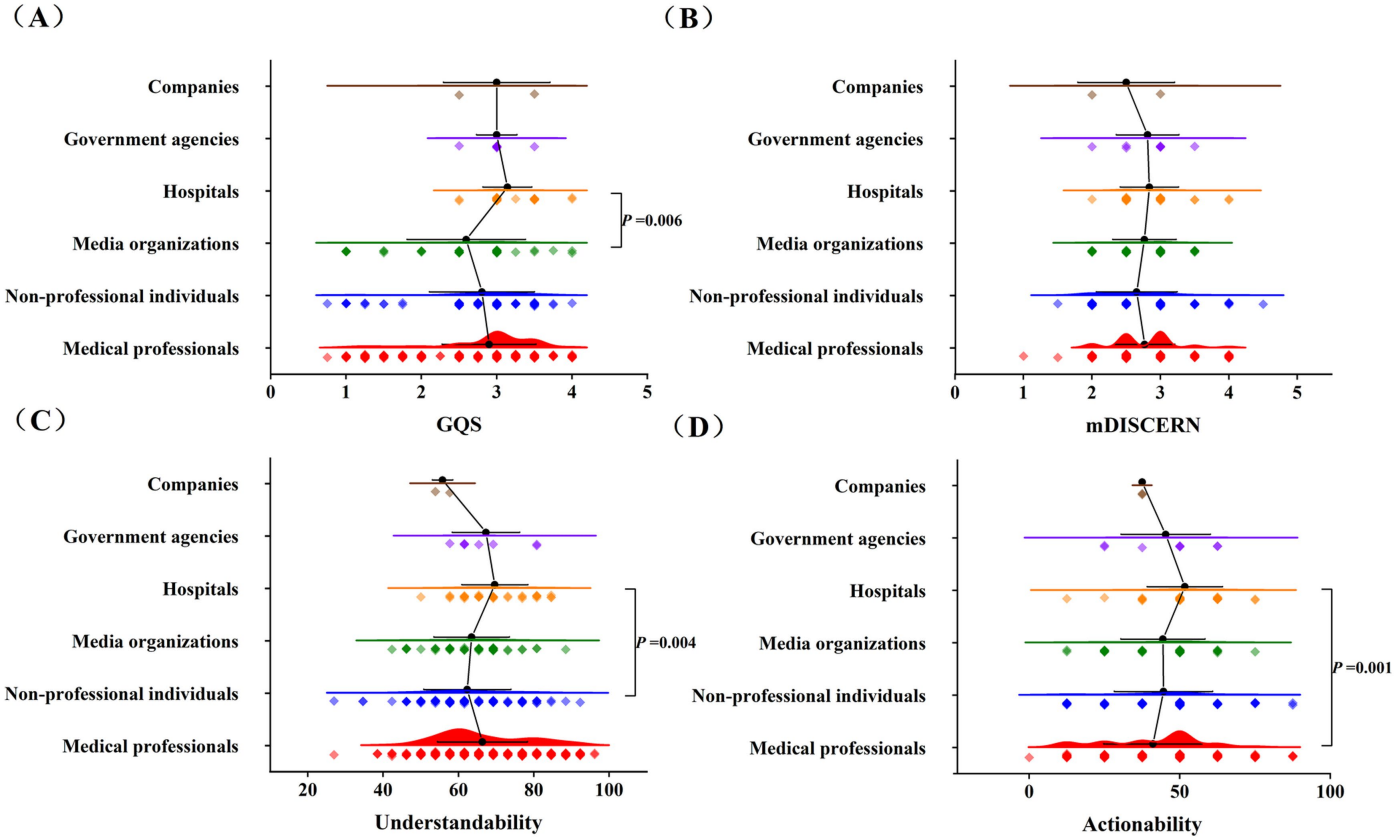
Comparison of quality scores of cerebrovascular diseases health science popularization short videos across different account types. **(A)** Comparison of GQS of cerebrovascular diseases health science popularization short videos for different account types; **(B)** Comparison of mDISCERN scores of cerebrovascular diseases health science popularization short videos for different account types; **(C)** Comparison of Understandability scores of cerebrovascular diseases health science popularization short videos for different account types; **(D)** Comparison of Actionability scores of cerebrovascular diseases health science popularization short videos for different account types.

Concurrently, we compared video quality across different account types. Results demonstrated that there were statistically significant differences in GQS scores, Understandability scores, and Actionability scores among videos posted by distinct creator accounts (*p* < 0.05; Figure 6). Figure 6A showed that for the GQS scores of different accounts, the score distribution of hospitals was relatively concentrated and high at 3 (IQR 3–3.5), compared with that of media organizations at 3 (IQR 2–3), and the difference between them was statistically significant (*p* = 0.006, *p* < 0.05). For the mDISCERN scores of different accounts, the scores were relatively dispersed, and there were no particularly significant differences between groups (Figure 6B). Figure 6C showed that in terms of Understandability scores, there was a difference between the score of hospitals at 69.23% (IQR 61.53–76.92%) and that of non-professional individuals at 61.53% (IQR 57.69%-69.23) (*p* = 0.004, *p* < 0.05). Figure 6D showed that in terms of Actionability scores, the score distribution of hospitals was relatively concentrated and high at 50% (IQR 50–62.5%), which was significantly different from that of medical professionals at 50% (IQR 25–50%) (*p* = 0.001, *p* < 0.05).

### 3.3 Key features influencing the quality of health science popularization short videos related to cerebrovascular diseases on TikTok and Kuaishou

The correlations between video features and quality assessment scores (mDISCERN, GQS, Understandability, and Actionability) were presented in Table 3. There were relatively strong correlations between video duration and mDISCERN score (*r* = 0.219, *p* < 0.001), GQS (*r* = 0.495, *p* < 0.001), Understandability score (*r* = 0.282, *p* < 0.001), and Actionability score (*r* = 0.361, *p* < 0.001). Additionally, there were moderate correlations between the Understandability score and likes (*r* = 0.153, *p* < 0.001), favorites (*r* = 0.169, *p* < 0.001), and shares (*r* = 0.163, *p* < 0.001). Similarly, likes (*r* = 0.221, *p* < 0.001), favorites (*r* = 0.217, *p* < 0.001), shares (*r* = 0.228, *p* < 0.001), and comments (*r* = 0.171, *p* < 0.001) showed a moderate correlation with the Actionability score. Furthermore, a positive correlation was observed between the publisher and the Actionability score (*r* = 0.123, *p* < 0.001).

**Table 3:** Correlation between the video features and video quality of cerebrovascular diseases health science popularization short videos.

Variable	r/p value	mDISCERN	GQS	Understandability	Actionability
Likes	*r*	0.030	0.053	0.153	0.221
	*p* value	0.361	0.110	<0.001***	<0.001***
Favorites	*r*	0.001	0.048	0.169	0.217
	*p* value	0.974	0.151	<0.001***	<0.001***
Shares	*r*	0.005	0.056	0.163	0.228
	*p* value	0.876	0.093	<0.001***	<0.001***
Comments	*r*	0.011	0.009	0.088	0.171
	*p* value	0.743	0.779	0.008*	<0.001***
Duration	*r*	0.219	0.495	0.282	0.361
	*p* value	<0.001***	<0.001***	<0.001***	<0.001***
Publishers	*r*	−0.036	−0.056	−0.035	0.123
	*p* value	0.281	0.092	0.294	<0.001***

*p < 0.05; ***p < 0.001; mDISCERN, modified DISCERN; GQS, Global Quality Score.

**Figure 7:**
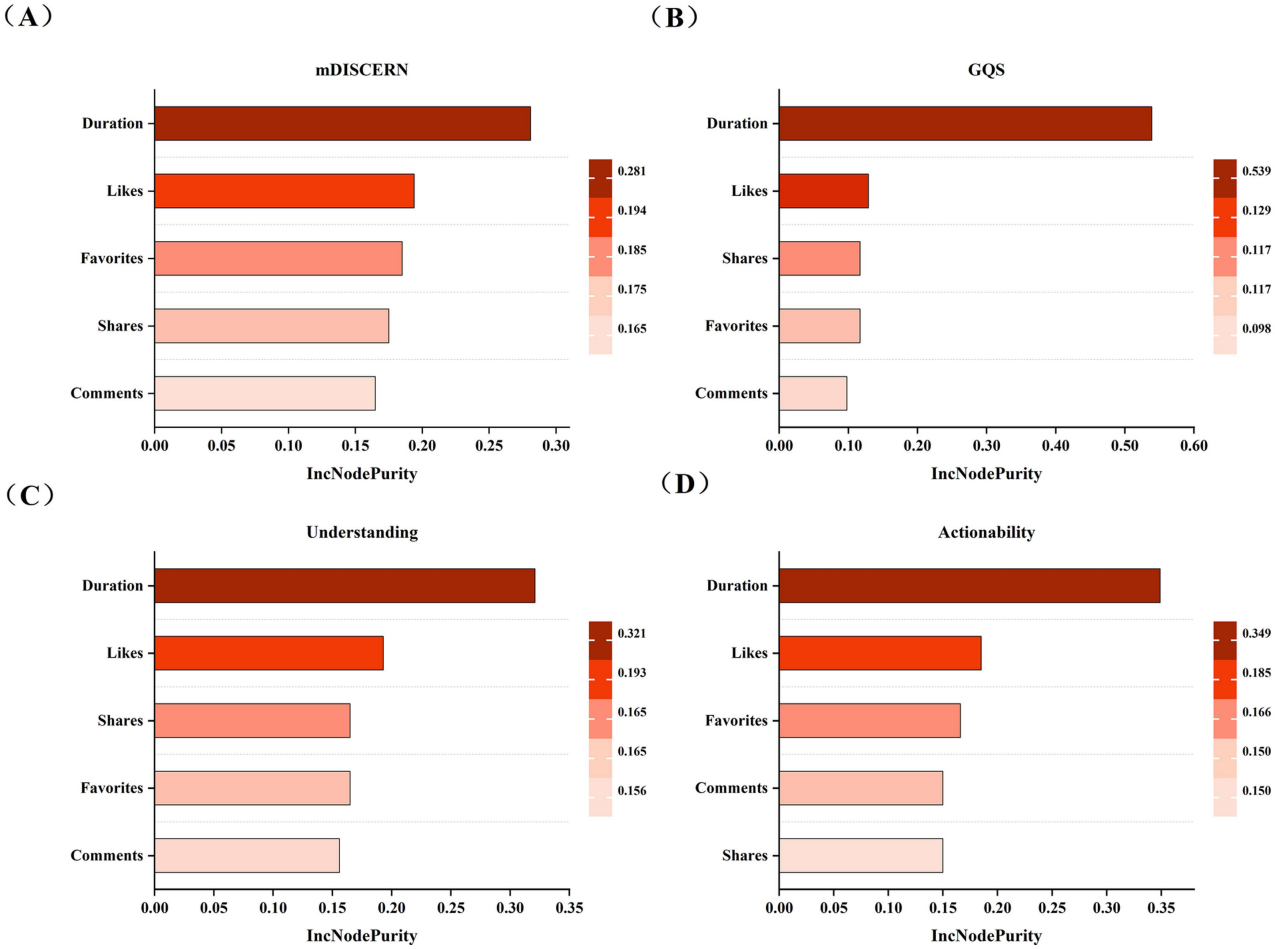
Weight of different video features in the random forest regression models. **(A)** Weight of different video features in the mDISCERN random forest regression model; **(B)** Weight of different video features in the GQS random forest regression model; **(C)** Weight of different video features in the Understandability random forest regression model; **(D)** Weight of different video features in the Actionability random forest regression model.

The results of the random forest regression model regarding the importance of video features to video quality were presented in Table 4. The four models demonstrated favorable fitting performance, with *R*^2^ values ranging from 0.862 to 0.903 and small error metrics such as MAE, MSE, and RMSE. Video duration emerged as the most critical feature contributing to all four models, with a weight proportion of 28.10% in the mDISCERN model (Figure 7A), 53.92% in the QRS model (Figure 7B), 32.11% in the Understandability model (Figure 7C), and 34.94% in the Actionability model (Figure 7D). The remaining features (likes, favorites, comments, and shares) had relatively lower weights, with slight variations in proportions across different models.

**Table 4:** Random forest regression model of the video features and video quality of cerebrovascular diseases health science popularization short videos.

Scores	*R^2^*	MAE	MSE	RMSE
mDISCERN	0.862	0.139	0.036	0.189
GQS	0.903	0.093	0.021	0.145
Understandability	0.877	3.726	22.145	4.706
Actionability	0.878	4.883	41.094	6.410

mDISCERN, modified DISCERN; GQS, Global Quality Score; R^2^ denotes the coefficient of determination; MAE denotes the Mean Absolute Error; MSE denotes Mean Squared Error; RMSE denotes for Root Mean Squared Error.

## 4 Discussion

### 4.1 Principal findings

In this study, a comprehensive evaluation was conducted on the quality of health science popularization short videos related to cerebrovascular diseases on two major Chinese short video platforms, TikTok and Kuaishou. Our results showed that there were more videos on TikTok, and these videos were more popular compared to videos on Kuaishou. We found that 88.17% of the videos on TikTok have a GQS score ≤ 3, 87.43% of the videos have an mDISCERN score ≤ 3, while 97.59% of the videos on Kuaishou have a GQS score ≤ 3, and 93.85% of the videos have an mDISCERN score ≤ 3. These results indicated that the overall quality of health science popularization short videos related to cerebrovascular diseases on TikTok and Kuaishou was moderate to low, which was consistent with the findings of Ge et al. (32) in their study on the quality evaluation of stroke-related videos. The results may be related to the entertainment-oriented nature of short-video platforms and the fact that health science popularization via new media is still in its initial stage. As commercial entertainment platforms, short video platforms are primarily profit-driven (33) and tend to prioritize the promotion of lighthearted, engaging content over scientifically rigorous materials. With the gradual surge in health-related videos on these platforms, some creators have resorted to oversimplifying or exaggerating scientific content to pursue traffic. This phenomenon is mainly due to the low appeal of complex and accurate scientific information to the general public, whereas false or misleading health information spreads more easily (15). Furthermore, there is no unified evaluation standard for the quality of health science popularization short videos until now, which makes it impossible for government agencies and new media platforms to exercise comprehensive control and supervision over the quality of health science popularization short videos.

### 4.2 Analysis of quality differences

This study found that health science popularization short videos corresponding to different keywords show differences in both quantity and quality. In terms of quantity, videos related to “cerebrovascular diseases” and “cerebral apoplexy” accounted for 48.2% in total, while those associated with “subarachnoid hemorrhage” only made up 8.3%. This discrepancy may be related to the varying levels of public attention toward different diseases and creators’ perceptions of the prevalence of these conditions. In terms of quality, videos about “transient ischemic attack” and “subarachnoid hemorrhage” obtained the lowest operability scores, indicating that the science popularization content for these two disease types is insufficient in guiding the public to take specific actions. Given that transient ischemic attack is an important warning sign of stroke (34), and subarachnoid hemorrhage is characterized by acute onset and high mortality (35), it is imperative to enhance the practical guidance of science popularization content for such diseases, so as to help the public identify key symptoms and take correct and timely responses.

We found that the majority of the health science popularization videos related to cerebrovascular diseases published on both platforms were created by medical professionals. This might be associated with national policies such as the “Healthy China 2030” Planning Outline. Encouraged by these policies, an increasing number of medical personnel are using short video platforms to disseminate health knowledge (23). Previous studies have shown that viewers preferred to viewing health science popularization videos from hospital accounts (11). This could be explained by the fact that the public hospitals gained more trust from the public. However, our findings indicated that the quality of health science popularization videos released by medical professionals was far from satisfactory. This may be related to the fact that they have limited time for content creation, lack video production skills, and have access to scarce training resources (36). Meanwhile, TikTok has prohibited medical professionals from engaging in live-streaming e-commerce, and its fan group function was also discontinued in 2023 (37). Such regulations may have diminished medical professionals’ motivation to produce high-quality health education videos. Our findings were inconsistent with the conclusions of Wang et al. (38). In their study evaluating the quality of stroke-related health science popularization short videos on TikTok, they found that videos created by medical professionals were significantly superior to those produced by other creators. This discrepancy might stem from the fact that they only selected the top 100 videos with the highest number of likes, whereas our study included a much larger sample of videos.

### 4.3 The impact of interaction metrics and algorithms

We found that the number of likes, favorites, comments and shares of health science popularization short videos on TikTok platform were higher than those on Kuaishou platform, indicating that users on TikTok platform had a higher level of participation and more interactions. This finding was consistent to previous research which found TikTok exhibited the highest engagement levels (39). Our study revealed that TikTok videos related to cerebrovascular diseases consistently outperformed those on Kuaishou across all evaluated metrics, including mDISCERN, GQS, Understandability, and Actionability scores. These findings aligned with previous studies which noted platform-specific variations in the quality of health information, such as the higher reliability of TikTok videos compared to other platforms in contexts like Liver cancer and acute pancreatitis (40, 41). This discrepancy may be associated with TikTok’s more sophisticated content dissemination algorithm (42), which takes interactive metrics as its core recommendation criteria. While prioritizing interactivity, TikTok’s algorithm also incorporates content quality into its evaluation, thus creating a positive feedback loop where high-quality health education videos generate greater user engagement and, in turn, achieve wider exposure. In addition, compared with Kuaishou, TikTok has implemented a more stringent content review mechanism. It has launched a Knowledge Creation Support Program and provides targeted traffic incentives for professional and valuable content.

However, it is worth noting that the general public tends to prefer videos with highly entertaining, sensational, or fragmented content (11, 43, 44). Such content usually generates higher real-time engagement, and the platform recommendation algorithms amplify their reach, thereby overshadowing the visibility of high-quality yet low-engagement health education videos. This phenomenon reflects the problem of short-video platforms’ over-reliance on interactive metrics, indicating that platforms could further optimize their recommendation mechanisms in the future to strike a balance between interactivity and content value. In addition, platforms could establish a dedicated review team for health-related content and implement a mandatory quality labeling system. They could offer traffic incentives for high-quality videos and facilitate collaborations between medical professionals and individual creators to co-produce content, thereby enabling professional and engaging high-quality videos to gain greater exposure through algorithmic recommendations.

### 4.4 Correlation between video quality and video features

The strong correlations between video duration and the four quality assessment metrics (mDISCERN, GQS, Understandability, and Actionability) observed in this study, coupled with its dominant weight in the Random Forest regression models (28.10 to 53.92%), highlighted video duration as a pivotal influencing feature of the quality of cerebrovascular disease health science popularization videos on TikTok and Kuaishou. Our finding was consistent to the previous studies which also found that video duration was a key predictor of the quality of the health science popularization short videos (45–47). This may be associated with the fact that shorter videos fail to convey sufficient information, whereas longer ones contain more comprehensive and complete health content. Therefore, publishers may appropriately extend the duration of health science popularization short videos.

Additionally, metrics such as likes, comments, favorites, and shares showed moderate correlations with Understandability and Actionability scores, and their weights in the Random Forest regression models were non-negligible. These features, to a certain extent, reflected the popularity of the videos (48). Our findings were consistent with Yeung’s research which reported that attention-deficit/hyperactivity disorder (ADHD) videos featuring personal experiences scored high in Understandability and were the most popular (49). This might be related to the fact that content with high actionability or easy understandability is likely to trigger higher engagement, and videos with higher engagement will be further disseminated and diffused under the recommendation of algorithms. Meanwhile, we found no correlations between video engagement metrics (likes, favorites, comments, and shares) and the mDISCERN and GQS scores of the videos. This research result was inconsistent with that of Gong et al. (50). This may be related to the bias resulting from the researchers’ manual search on the platform and the inclusion of short videos with relatively high user engagement.

### 4.5 Strengths and limitations

The advantages of our research were as follows. First, we selected TikTok and Kuaishou, the two most representative short video platforms in China. Compared with other studies, this approach overcomes the limitations of single-platform research. These platforms, with the largest user bases in China and high user loyalty, have become an indispensable part of the daily lives of internet users. Second, we employed web crawler technology to collect videos. Unlike other studies that only select the top 100 videos based on the platform’s comprehensive rankings, the data collected through crawling technology is more comprehensive, making the research objects more representative. Third, we utilized multiple evaluation tools (GQS, mDISCERN, and PEMAT-A/V) to assess the quality of cerebrovascular health science popularization videos, enabling a comprehensive and multi-dimensional measurement that reflected distinct aspects of content quality. This multi-tool approach avoided the limitations of single-instrument assessments, and thus enhanced the robustness and validity of our evaluations. Furthermore, we conducted a further analysis of the correlations between video quality and video features including account type, video duration, likes, favorites, comments, and shares. The aim was to identify the key factors influencing video quality, thereby providing video creators with better creative ideas.

However, our research also had some limitations. First, the selection of video keywords in this study may not cover all relevant expressions of cerebrovascular diseases, resulting in a certain degree of selection bias. Future research could expand the scope of keywords by incorporating natural language processing (NLP) techniques. The algorithms of short video platforms are dynamic, leading to temporal variations in video exposure and visibility. This study could not fully eliminate the confounding effects of algorithmic fluctuations on sample selection, which imposes certain constraints on the representativeness and stability of the study sample. Second, despite the fact that both raters received relevant training prior to scoring, the research tools employed were relatively subjective. Coupled with visual fatigue caused by raters viewing a large number of videos, this might result in relatively subjective scoring outcomes. Future research could adopt more objective evaluation methods to assess the quality of health education videos. Third, our study is limited to the data of health science popularization videos, and does not incorporate the behavioral data of video audiences. Future research could integrate the behavioral data of video audiences to further explore the relationship between health science popularization videos and audience behaviors.

## 5 Conclusion

This study evaluated the quality of cerebrovascular diseases health science popularization videos on TikTok and Kuaishou. The overall quality of videos on both TikTok and Kuaishou platforms was generally unsatisfactory, and in comparison, the quality of videos on TikTok was higher than that on Kuaishou. We found that video quality varied significantly across different account entities. Engagement metrics were closely associated with video quality. Video duration emerged as a key factor influencing video quality. This study can provide data support for platforms to establish video quality supervision mechanisms and for governments to improve the standardization of science popularization content. It can also help the public obtain high-quality health information from short-video platforms, thereby enhancing public health literacy.

## Data Availability

The raw data supporting the conclusions of this article will be made available by the authors, without undue reservation.

## References

[1] Cheng Y Lin Y Shi H Cheng M Zhang B Liu X . Projections of the Stroke Burden at the Global, Regional, and National Levels up to 2050 Based on the Global Burden of Disease Study 2021. J Am Heart Assoc. (2024). 13:e036142 doi: 10.1161/jaha.124.036142 PMC11681572 39575720

[2] GBD 2021 Diseases and Injuries Collaborators. Global incidence, prevalence, years lived with disability (YLDs), disability-adjusted life-years (DALYs), and healthy life expectancy (HALE) for 371 diseases and injuries in 204 countries and territories and 811 subnational locations, 1990-2021: a systematic analysis for the global burden of disease study 2021. Lancet. (2024) 403:2133–61. doi: 10.1016/s0140-6736(24)00757-8, 38642570 PMC11122111

[3] Feigin VL Owolabi MO. Pragmatic solutions to reduce the global burden of stroke: a world stroke organization-lancet neurology commission. Lancet Neurol. (2023) 22:1160–206. doi: 10.1016/s1474-4422(23)00277-6, 37827183 PMC10715732

[4] National Health Commission of the People’s Republic of China. Guidelines for the prevention and treatment of cerebrovascular diseases (2024 edition). (2024). Available online at: https://www.nhc.gov.cn/ylyjs/zcwj/202412/ba037e931fff4870930f65ff667ea9ed.shtml (accessed [15 December, 2025]).

[5] Mengjie H Leixiao Z Pu G Xincheng H Yujia W Yibo w. Perceived needs of health education and associated factors among community-dwelling residents. Chin Gen Pract. (2023) 26:426–33. doi: 10.12114/j.issn.1007-9572.2022.0632.

[6] Li J Song J Zhu XL Chen MF Huang XF. Analysis of status quo and influencing factors for health-promoting lifestyle in the rural populace with high risk of cardiovascular and cerebrovascular diseases. BMC Cardiovasc Disord. (2023) 23:118. doi: 10.1186/s12872-023-03129-7, 36890439 PMC9996853

[7] Feigin VL Brainin M Norrving B Martins SO Pandian J Lindsay P. World stroke organization: global stroke fact sheet 2025. Int J Stroke. (2025) 20:132–44. doi: 10.1177/17474930241308142, 39635884 PMC11786524

[8] Jin C Jun L Zu W. Analysis of the correlation between article content and user feedback on hospital wechat public numbers. Mod Hosp J. (2025) 25:840–4. doi: 10.3969/j.issn.1671-322X.2025.06.005

[9] Yang S Zhan J Xu X. Is TikTok a high-quality source of information on thyroid cancer? Endocrine. (2023) 81:270–6. doi: 10.1007/s12020-023-03332-8, 36840912

[10] Center CINI. 55th statistical report on internet development in China: China internet network information center (2025). Available online at: https://www.cnnic.net.cn/n4/2025/0117/c88-11229.html (Accessed July 25, 2025).

[11] Xiao L Min H Wu Y Zhang J Ning Y Long L. Public's preferences for health science popularization short videos in China: a discrete choice experiment. Front Public Health. (2023) 11:1160629. doi: 10.3389/fpubh.2023.1160629, 37601206 PMC10436607

[12] Yan Z Jing G. Research on the evolution mechanism of users' health anxiety relief through health science popularization short videos. Libr Inf Serv. (2025) 69:96–106. doi: 10.13266/j.issn.0252-3116.2025.06.008

[13] van Kampen K Laski J Herman G Chan TM. Investigating COVID-19 vaccine communication and misinformation on TikTok: cross-sectional study. JMIR Infodemiol. (2022) 2:e38316. doi: 10.2196/38316, 36338548 PMC9620417

[14] Li HO Bailey A Huynh D Chan J. YouTube as a source of information on COVID-19: a pandemic of misinformation? BMJ Glob Health. (2020) 5:e002604. doi: 10.1136/bmjgh-2020-002604, 32409327 PMC7228483

[15] Suarez-Lledo V Alvarez-Galvez J. Prevalence of health misinformation on social media: systematic review. J Med Internet Res. (2021) 23:e17187. doi: 10.2196/17187, 33470931 PMC7857950

[16] Loeb S Reines K Abu-Salha Y French W Butaney M Macaluso JN Jr . Quality of bladder cancer information on YouTube. Eur Urol. (2021) 79:56–9. doi: 10.1016/j.eururo.2020.09.014, 33010986

[17] John JN Gorman S Scales D Gorman J. Online misleading information about women's reproductive health: a narrative review. J Gen Intern Med. (2025) 40:1123–31. doi: 10.1007/s11606-024-09118-6, 39511120 PMC11968640

[18] Starvaggi I Dierckman C Lorenzo-Luaces L. Mental health misinformation on social media: review and future directions. Curr Opin Psychol. (2024) 56:101738. doi: 10.1016/j.copsyc.2023.101738, 38128168

[19] Lai Y Liao F He Z Lai W Zhu C Du Y . The status quo of short videos as a health information source of *Helicobacter pylori*: a cross-sectional study. Front Public Health. (2023) 11:1344212. doi: 10.3389/fpubh.2023.1344212, 38259733 PMC10800962

[20] QuestMobile. 2025 China Mobile internet spring report. (2025). Available online at: https://www.questmobile.com.cn/research/report/1919961024158601218 (accessed [25 July, 2025]).

[21] Kanner J Waghmarae S Nemirovsky A Wang S Loeb S Malik R. TikTok and YouTube videos on overactive bladder exhibit poor quality and diversity. Urol Pract. (2023) 10:493–500. doi: 10.1097/upj.0000000000000423, 37347790

[22] Li Z Yan C Lyu X Li F Zeng R. Assessing quality and reliability of online videos on tachycardia: a YouTube video-based study. BMC Public Health. (2024) 24:2620. doi: 10.1186/s12889-024-20062-2, 39334090 PMC11438393

[23] Mao T Zhao X Jiang K Yang J Xie Q Fu J. Evaluation of TikTok videos on acute pancreatitis: content quality and reliability analysis. BMC Public Health. (2024) 24:1216. doi: 10.1186/s12889-024-18708-2, 38698404 PMC11067236

[24] Bernard A Langille M Hughes S Rose C Leddin D van Veldhuyzen Zanten S. A systematic review of patient inflammatory bowel disease information resources on the world wide web. Am J Gastroenterol. (2007) 102:2070–7. doi: 10.1111/j.1572-0241.2007.01325.x, 17511753

[25] Singh AG Singh S Singh PP. YouTube for information on rheumatoid arthritis--a wakeup call? J Rheumatol. (2012) 39:899–903. doi: 10.3899/jrheum.111114, 22467934

[26] Wang G Kuang J Qi Y Li J. Information quality of videos related to adolescent depression on social media platforms: a comparative study of TikTok and BiliBili. Front Public Health. (2025) 13:1663977. doi: 10.3389/fpubh.2025.1663977, 41346755 PMC12672318

[27] Wang S Jia S Su Y Cheng R. Videos on YouTube, Bilibili, TikTok as sources of medical information on Hashimoto's thyroiditis. Front Public Health. (2025) 13:1611087. doi: 10.3389/fpubh.2025.1611087, 41170477 PMC12568602

[28] Chen Y Wang Q Huang X Zhang Y Li Y Ni T. The quality and reliability of short videos about thyroid nodules on BiliBili and TikTok: cross-sectional study. Digit Health. (2024) 10:20552076241288831. doi: 10.1177/20552076241288831, 39381823 PMC11459542

[29] Charnock D Shepperd S Needham G Gann R. DISCERN: an instrument for judging the quality of written consumer health information on treatment choices. J Epidemiol Community Health. (1999) 53:105–11. 10396471 10.1136/jech.53.2.105 PMC1756830

[30] Min L Yang Z Bingyan L Shuo Y Wenjuan Y X L. Progress on quality assessment tools for health science popularization videos. Chinese General Practice. (2026). https://link.cnki.net/urlid/13.1222.R.20250324.0916.008

[31] Shoemaker SJ Wolf MS Brach C. Development of the patient education materials assessment tool (PEMAT): a new measure of understandability and actionability for print and audiovisual patient information. Patient Educ Couns. (2014) 96:395–403. doi: 10.1016/j.pec.2014.05.027, 24973195 PMC5085258

[32] Ge R Dai H Gong C Xia Y Wang R Xu J. The quality and reliability of online videos as an information source of public health education for stroke prevention in mainland China: electronic media-based cross-sectional study. JMIR Infodemiol. (2025) 5:e64891. doi: 10.2196/64891, 40690658 PMC12303359

[33] Linlin W Yuan R. Research on business model innovation of Douyin platform from the perspective of value co-creation. China J Commer. (2024) 33:126–30. doi: 10.19699/j.cnki.issn2096-0298.2024.23.126

[34] Haixu Z Beizhu Y Yusheng L Yuming X Yuang G. Health economics evaluation of stroke screening strategies based on a decision tree-Markov model. Med Soc. (2026) 39:128–35. doi: 10.13723/j.yxysh.2026.01.017

[35] Kleindorfer DO Towfighi A Chaturvedi S Cockroft KM Gutierrez J Lombardi-Hill D. 2021 guideline for the prevention of stroke in patients with stroke and transient ischemic attack: a guideline from the American Heart Association/American Stroke Association. Stroke. (2021) 52:e364–467. doi: 10.1161/str.0000000000000375, 34024117

[36] Dalton C Sarwar Z Garwe T Hunter CJ. Evaluating perceptions of social media professionalism by healthcare workers. Digit Health. (2026) 12:1–12. doi: 10.1177/20552076251411281, 41551640 PMC12804638

[37] Kong D Qi J. From clinics to clicks: investigating the motivations and competency framework of healthcare professionals on social media. Digit Health. (2025) 11:20552076251377845. doi: 10.1177/20552076251377845, 40949667 PMC12432320

[38] Wang Z Hu C Zhou B Wan M. Quality evaluation of stroke-related information on TikTok: a cross-sectional study. Sci Rep. (2025) 16:1843. doi: 10.1038/s41598-025-31464-6, 41388070 PMC12804945

[39] Liu Z Chen Y Lin Y Ai M Lian D Zhang Y. YouTube/ Bilibili/ TikTok videos as sources of medical information on laryngeal carcinoma: cross-sectional content analysis study. BMC Public Health. (2024) 24:1594. doi: 10.1186/s12889-024-19077-6, 38877432 PMC11177428

[40] Zheng S Tong X Wan D Hu C Hu Q Ke Q. Quality and reliability of liver Cancer-related short Chinese videos on TikTok and Bilibili: cross-sectional content analysis study. J Med Internet Res. (2023) 25:e47210. doi: 10.2196/47210, 37405825 PMC10357314

[41] Zhu YC Du RC Gao J Lu NH Zhu Y Hu Y. Youtube and TikTok as sources of information on acute pancreatitis: a content and quality analysis. BMC Public Health. (2025) 25:1446. doi: 10.1186/s12889-025-22738-9, 40247311 PMC12004688

[42] Liang Y Xia J Huo W Liu B Wang Z Ding Y. Video quality assessment and analysis of gastroesophageal reflux disease on TikTok and Bilibili: cross-sectional study. J Multidiscip Healthc. (2024) 17:5927–39. doi: 10.2147/jmdh.S485781, 39678716 PMC11646458

[43] Davis LS León B Bourk MJ Finkler W. Transformation of the media landscape: infotainment versus expository narrations for communicating science in online videos. Public Underst Sci. (2020) 29:688–701. doi: 10.1177/0963662520945136, 32729396

[44] Wenhua H Chunqing L. The impact of fragmentation of short video on pleasure and functional experience ambidexterity —based on information processing theory. Econ Manag. (2026):1–15. Available online at: https://link.cnki.net/urlid/13.1032.F.20260115.1129.002

[45] Ming S Han J Yao X Guo X Guo Q Lei B. Myopia information on TikTok: analysis factors that impact video quality and audience engagement. BMC Public Health. (2024) 24:1194. doi: 10.1186/s12889-024-18687-4, 38685020 PMC11057166

[46] Cai QY Tang J Meng SZ Sun Y Lan X Liu TH. Quality assessment of videos on social media platforms related to gestational diabetes mellitus in China: a cross-section study. Heliyon. (2024) 10:e29020. doi: 10.1016/j.heliyon.2024.e29020, 38617917 PMC11015130

[47] Sun F Zheng S Wu J. Quality of information in gallstone disease videos on TikTok: cross-sectional study. J Med Internet Res. (2023) 25:e39162. doi: 10.2196/39162, 36753307 PMC9947761

[48] Guan JL Xia SH Zhao K Feng LN Han YY Li JY . Videos in short-video sharing platforms as sources of information on colorectal polyps: cross-sectional content analysis study. J Med Internet Res. (2024) 26:e51655. doi: 10.2196/51655, 39470708 PMC11558218

[49] Yeung A Ng E Abi-Jaoude E. TikTok and attention-deficit/hyperactivity disorder: a cross-sectional study of social media content quality. Can J Psychiatr. (2022) 67:899–906. doi: 10.1177/07067437221082854, 35196157 PMC9659797

[50] Gong X Chen M Ning L Zeng L Dong B. The quality of short videos as a source of coronary heart disease information on TikTok: cross-sectional study. JMIR Form Res. (2024) 8:e51513. doi: 10.2196/51513, 39226540 PMC11408897

