# Supplementary material for: Quality evaluation of health science popularization short videos related to cerebrovascular diseases on popular short video platforms in China: cross-sectional study
Source: Front Public Health. 2026 Feb 19;14:1764220. doi: 10.3389/fpubh.2026.1764220 (PMC12960548; doi:10.3389/fpubh.2026.1764220)
Supplement: Supplementary file 1 [file Data_Sheet_1.docx]

**Supplementary Table 1 Global Quality Scale**

| **Item** | **Score** |
| --- | --- |
| Poor quality, poor flow, most information missing, not helpful for patients | 1 |
| Generally poor, some information given but of limited use to patients | 2 |
| Moderate quality, some important information is adequately discussed | 3 |
| Good quality good flow, most relevant information is covered, useful for patients | 4 |
| Excellent quality and excellent flow, very useful for patients | 5 |

**Supplementary Table 2 Modified DISCERN reliability tool**

| **Item** |
| --- |
| 1. Are the aims clear and achieved? |
| 2. Are the sources of information reliable? |
| 3. Is the information balanced and unbiased? |
| 4. Are additional resources to information provided? |
| 5. Does the video address areas of controvesy/uncertainty? |

**Supplementary Table 3 PEMAT Tool for Audiovisual Materials (PEMAT-A/V)**

| **Item** |
| --- |
| **Understandability** |
| 1.The material makes its purpose completely evident |
| 2. The material uses common, everyday language. |
| 3. Medical terms are used only to familiarize audience with the terms. When used, medical terms are defined. |
| 4. The material uses the active voice. |
| 5. The material breaks or "chunks" information into short sections. |
| 6. The material’s sections have informative headers. |
| 7. The material presents information in a logical sequence. |
| 8. The material provides a summary. |
| 9. The material uses visual cues (e.g., arrows, boxes, bullets, bold, larger font, highlighting) to draw attention to key points. |
| 10. Text on the screen is easy to read. |
| 11. The material allows the user to hear the words clearly (e.g., not too fast, not garbled). |
| 12. The material uses illustrations and photographs that are clear and uncluttered. |
| 13. The material uses simple tables with short and clear row and column headings. |
| **Actionability** |
| 14. The material clearly identifies at least one action the user can take. |
| 15. The material addresses the user directly when describing actions. |
| 16. The material breaks down any action into manageable, explicit steps. |
| 17. The material explains how to use the charts, graphs, tables, or diagrams to take actions. |

**Supplementary Table 4 Quantity and Quality of Videos by Keyword**

| **Keywords** | **Qu****antity (Proportion)** | **Median (IQR)** | | | |
| --- | --- | --- | --- | --- | --- |
|  |  | **mDISCERN** | **GQS** | **Understandability** | **Actionability** |
| **cerebrovascular disease** | 241(26.3%) | 3.00(2.50,3.00) | 3.00(3.00,3.00) | 65.38(57.69,80.77) | 50(25,50) |
| **cerebral apoplexy** | 201(22.0%) | 3.00(2.50,3.00) | 3.00(3.00,3.00) | 61.54(53.85,76.92) | 50(37.5,50) |
| **stroke** | 78(8.5%) | 3.00(2.00,3.00) | 3.00(2.50,3.00) | 61.54(53.85,65.38) | 50(50,50) |
| **cerebral infarction** | 135(14.8%) | 3.00(2.50,3.00) | 3.00(3.00,3.00) | 61.54(53.85,76.92) | 50(37.5,50) |
| **transient ischemic attack** | 80(8.7%) | 3.00(3.00,3.00) | 3.00(3.00,3.00) | 57.69(53.85,65.38) | 25(25,50) |
| **intracerebral hemorrhage** | 104(11.4%) | 3.00(3.00,3.00) | 3.00(3.00,3.00) | 61.54(53.85,65.38) | 50(25,50) |
| **subarachnoid hemorrhage** | 76(8.3%) | 3.00(2.75,3.00) | 3.00(3.00,3.00) | 57.69(53.85,65.38) | 25(25,50) |
